# Modification of the height of a weight drop traumatic brain injury model that causes the formation of glial scar and cognitive impairment in rats

**DOI:** 10.1186/s12883-023-03494-y

**Published:** 2023-12-15

**Authors:** Donny Wisnu Wardhana, Hendy Setyo Yudhanto, Wibi Riawan, Husnul Khotimah, Happy Kurnia Permatasari, Tommy Alfandy Nazwar, Nurdiana Nurdiana

**Affiliations:** 1 Doctoral Program in Medical Sciences, Faculty of Medicine, Universitas Brawijaya, Malang, Indonesia; 2 Department of Anatomy Pathology, Faculty of Medicine, Universitas Brawijaya, Malang, Indonesia; 3 Department of Biomolecular Biochemistry, Faculty of Medicine, Universitas Brawijaya, Malang, Indonesia; 4 Department of Pharmacology, Faculty of Medicine, Universitas Brawijaya, Malang, Indonesia; 5 Department of Surgery, Faculty of Medicine, Universitas Brawijaya/Saiful Anwar General Hospital, Malang, Indonesia

**Keywords:** Traumatic brain Injury, Weight Drop Model, Glial scar, Cognitive impairment

## Abstract

**Objective:**

Traumatic brain injury (TBI) is a chronic, progressive condition associated with permanent disabilities, particularly cognitive impairments. Glial scar formation following TBI is considered a contributing factor to these persistent disabilities. Currently, limited research exists on pharmacological interventions targeting glial scar prevention that require a standard weight drop TBI model for glial scar formation. Since there is no established standard TBI model for glial scar formation, this study aims to validate and modify the height of the weight drop model to identify glial scar formation and cognitive impairments.

**Methods:**

Fifteen male Sprague Dawley rats were randomly divided into sham, WD1, and WD2 groups. The weight drop model with a 10 g load was applied to the right exposed brain of the rats from a height of 5 cm (WD1) and 10 cm (WD2) using a modified Feeney’s weight drop device. Cognitive impairments were confirmed using the novel object recognition (NOR) test with ethovision software on day 15. Subsequently, the rats were decapitated on day 16, and GFAP immunohistochemical staining was performed to confirm the presence of glial scarring.

**Results:**

The WD1 and WD2 groups exhibited a significant increase in glial scar formation compared to the sham group, with the WD2 group resulting in even more pronounced glial scar formation. Only the WD2 model caused statistically significant cognitive damage. The negative correlation coefficient indicates that an increase in GFAP + cells will decrease the cognitive function.

**Conclusion:**

Modification of the height of the weight drop model, by dropping a weight of 10 g from a height of 10 cm (WD2 group) onto the right brain exposed of the rat has been proven to induce the formation of a glial scar and cognitive impairment.

**Supplementary Information:**

The online version contains supplementary material available at 10.1186/s12883-023-03494-y.

## Introduction

Over the past three decades, animal models have been developed to mimic various aspects of traumatic brain injury (TBI) that occur in humans to better understand the underlying pathophysiology and search for effective therapeutic interventions [1]. However, the complex mechanisms and clinical situations of TBI in humans present challenges in replicating these TBI models in animal studies. Although larger animals have sizes and physiologies closer to humans, rodent animals are mostly used in TBI research due to their low cost, small size, and standardized outcome measures. Among various TBI models in rodents, three models are widely used: the weight drop model, fluid percussion injury, and controlled cortical impact [2].

The weight drop model has been extensively used in the literature since the early 1990s and remains a dominant model in use today [3, 4]. The weight drop model was initially developed by Feeney to provide an impact on the intact dura mater through a craniotomy, resulting in cortical contusions, lesion hemorrhage, blood-brain barrier disruption, immune cell infiltration, and glial cell activation according to the severity of the injury [2]. The weight drop model was further developed with shorter drop heights to induce concussion, cell death, short-term edema, and long-term cognitive deficits [2].

TBI is a chronic progressive disease that can have long-term impacts on physical, cognitive, behavioral, and emotional functioning. Cognitive impairments can manifest as attention deficits, decreased information processing abilities, confusion, and disorientation [5]. Various studies have demonstrated persistent cognitive impairments following TBI using the close head weight drop model in experimental rats [6]. Assessment of cognitive function in experimental rats can be performed using various tools to evaluate memory retention and learning ability in rats after injury [7].

Axonal damage and the lack of axon regeneration within the central nervous system are believed to be the primary reasons for the persistence of symptoms following injuries to the central nervous system. Central nervous system neurons can regenerate their axons if they are in a permissive environment. The central nervous system does not provide a permissive environment for axon regeneration. The formation of a glial scar, which includes inhibitory molecules and secreted proteins, inhibit axon regeneration. In addition to the glial scar, there are other factors suspected to inhibit regeneration, such as semaphorins and myelin-associated glycoproteins. Apart from the non-permissive axon regeneration environment, the central nervous system does not express pro-regenerative genes as seen in the peripheral nervous system [8].

Persistent cognitive impairments following TBI are caused by the formation of glial scars, which act as a physical and biochemical barrier to brain recovery in the later stages. Glial scars are formed by reactive astrocytes or astrogliosis, creating a nonpermissive environment that inhibits axonal regeneration. This condition is further exacerbated by regenerative failure due to the inadequate intrinsic capacity of neurons in the central nervous system (CNS) to repair injured axons [9].

GFAP expression has become a prototypical marker for the identification of astrocytes in immunohistochemistry. Pathophysiologically, when there is a change in the local biochemical environment following TBI, danger signals induce structural and functional changes in astrocytes, including hypertrophy and increased GFAP expression. This leads to astrocyte activation (reactive astrogliosis) [10, 11].

At the current stage, there have been limited studies on the weight drop model of TBI in experimental rats that can induce cognitive impairments and glial scar formation. In previous studies, researchers found two studies that demonstrated cognitive impairments and astrogliosis formation in rats induced with the weight drop model of TBI. The first study was conducted by Xu et al., [12] who dropped a 200 g weight from a height of 2.5 cm onto the exposed skull of mice. The second study was conducted by Chen et al., [13] on rats in which the brain was exposed by dropping a weight of 10 g from a height of 5 cm. The brain exposed method has an advantage over skull exposed as it ensures the visual formation of cortical contusion as the cause of glial scar formation. However, the disadvantage is that the weight drop treatment is more challenging and requires an expert to perform trepanation on the rat’s head, a longer time is required, more equipment is needed, and there is a higher risk of experimental animal mortality. Previous studies did not clearly define the glial scar and only observed the formation of astrogliosis, which is an early stage of glial scar formation. Therefore, the researchers aim to validate the weight drop model methods and modify the height of the weight drop model to achieve a glial scar formation accompanied by persistent cognitive impairment using the novel object recognition (NOR) test.

## Methods

### Animal

We conducted research on rats and obtained ethical approval from the Research Ethics Commission of the Faculty of Medicine, Universitas Brawijaya No. 246/EC/KEPK-S3/11/2022. A total of 15 male Sprague Dawley rats, aged three months and weighing 250–350 g with an average weight of 280 g per group, were obtained from Universitas Gadjah Mada, Yogyakarta, Indonesia. All rats were randomly housed in individual large cages under constant temperature (23 ± 2 °C), constant humidity (55 ± 5%), and a 12-hour light/dark cycle, and provided with standard food and water ad libitum. The rats were allowed to acclimate in the laboratory for 7 days prior to the experimental procedures. Each rat was used only once in order to minimize suffering. Rats that died, developed postoperative wound infections, or suffered from conditions unrelated to the experiment were excluded from the study. An increased weight drop can cause more serious damage to the rat’s brain, increasing the risk of bleeding, complications, and post-treatment mortality before evaluated for 14 days. The conditions during the procedures were standardized and consistent across all groups. The assessment of outcomes was performed by experts blinded to the treatment group of the animals,

### Animal model establishment and group

The sample size for this research was calculated using the resource question method, employing three sample groups with a 10% dropout rate, resulting in five samples in each group. Fifteen rats were randomly selected based on the computer-generated randomization results and divided into three groups: sham group, WD1 group, and WD2 group.

- Sham group: The rats did not receive any weight drop model, but cognitive testing was conducted using the NOR test on the first day. On day 16 the rats were decapitated, and the next day immunohistochemical staining was performed.
- Weight Drop 1 (WD1) group: On the first day, the rats received a weight drop model with a 10 g weight dropped from a height of 5 cm. Cognitive testing using the NOR test was conducted on day 15. On day 16 the rats were decapitated and immunohistochemical staining was performed.
- Weight Drop 2 (WD2) group: On the first day, the rats received a weight drop model with a 10 g weight dropped from a height of 10 cm. Cognitive testing using the NOR test was conducted on day 15. On day 16 the rats were decapitated and immunohistochemical staining was performed.

### Weight drop device

**Fig. 1:**
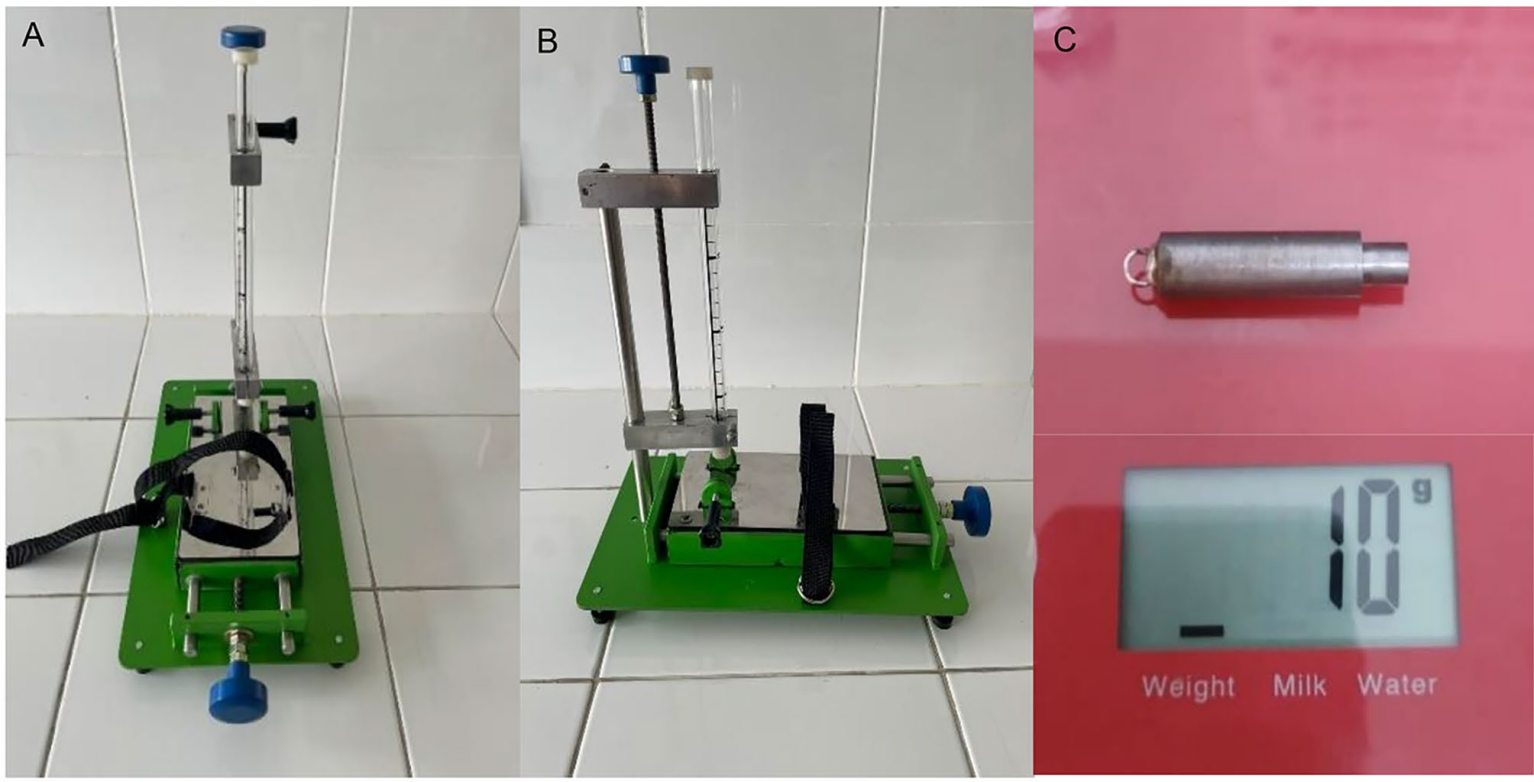
The weight drop model uses a transparent load delivery tube measuring 20 cm in length to facilitate the modification of the height from which the weight is dropped, creating a traumatic brain injury model in rats. (**A**) Front view of the device; (**B**) Side view of the device; (**C**) A weight of 10 g was dropped on the exposed brain of a rat

The weight drop device is an innovative form of the Feeney’s weight drop model. It consists of a metal flatbed to hold the rats, equipped with a head pin to immobilize the rat’s head and a seatbelt to secure the rat’s body during the procedure. At the front end of the flat bed, there is a projectile support holder in the form of a tube connected to a transparent glass tube measuring 25 cm in length. The transparent glass tube was equipped with a long ruler with centimeter units to drop the weight onto the rat’s head from a specific height. The glass tube can be rotated 360 degrees to adjust the position where the weight is dropped onto the rat’s head according to the research requirements (Fig. 1).

### TBI models (weight drop)

The rats in each injured group were randomly selected based on the results of randomization using a computer application. The rats were given prophylactic antibiotics Cefazolin sodium prior the injury at a dosage of 33 mg/kg, and intraperitoneal anesthesia with ketamine and xylazine. Rats were not administered analgesia as it could influence the nerve inflammation process in the formation of glial scar. Rats fixed on a stereotactic device and the rat’s scalp was shaved and cleaned with 10% povidone iodine solution. The aseptic technique was employed according to the surgical procedure. Subsequently, a 10 mm diameter craniotomy was performed on the right side of the brain, 2.5 mm posterior to the bregma, and 3 mm lateral to the midline in all groups. The dural incision was performed using a number 11 scalpel. A 10-gram weight with an end diameter of approximately 2.5 mm was dropped onto the brain surface from a height of 5 cm (WD1 group) or 10 cm (WD2 group), and the piston was allowed to compress the tissue maximally by 2.5 mm. This procedure resulted in moderate brain injury with small focal lesions, clearly visible contusions, and good recovery in the animals. The skin was sutured with interrupted 6 − 0 silk sutures. Subsequently, the rats returned to their respective individual small cages and maintained at room temperature (23 ± 1 °C) for 14 days. Each cage had an identical appearance and was assigned a code known only to the researcher to distinguish the groups.

### Cognitive test: novel object recognition test

The objects were arranged to contrast with the rat and ensure that they did not move due to the rat’s activity. Observations were conducted during daylight hours with the room lighting set to a diffused level of about 20 lx. Excessive brightness in the room could cause the arena to reflect light, resulting in bias with the color of the rats. On the other hand, overly dark lighting would make it difficult to observe the video recordings on EthoVision. The temperature and humidity of the arena were set to match the conditions in the cages. The camera was positioned above the arena to obtain an optimal view of exploration. During testing, the room was ensured to be quiet to reduce the anxiety of the rats during the experiments. The environmental conditions during the experiments were kept consistent across all groups, and the observations were conducted by an observer who was blinded to the groups.

First, the rats underwent habituation, which involved placing them in an empty arena for 5 min to familiarize themselves with the environment. After 5 min, the rats were removed from the arena and placed back in their cages. Subsequently, the arena was cleaned using 70% ethanol and stored. Twenty-four hours after habituation, the rats underwent training. Rats were randomly selected and placed back into the arena where two identical objects were positioned in opposite quadrants of the arena. The rats were placed in the center of the arena at an equal distance from the two identical objects and allowed to explore the objects for 5 min. After completion, the animals were returned to their cages, while the arena and objects were cleaned using 70% ethanol.

After 1 to 2 h from the training phase, the rats underwent testing. One of the objects in the arena was replaced with a novel object and positioned in the same location as in the training phase. Rats were randomly selected and placed back into the arena. The rats were given some time to familiarize themselves with the arena, and then their behavior was recorded to assess exploration toward the two objects for a duration of 5 min. After the testing phase concluded, the animals were returned to their cages, while the arena and objects were cleaned again using 70% ethanol. Exploration was defined as sniffing or touching with the nose and/or front paws within a distance of 2–3 cm around the object or climbing on the object. Cognitive impairment was evaluated based on discrimination index calculations ($\frac{\text{t}\text{i}\text{m}\text{e}\, \text{s}\text{p}\text{e}\text{n}\text{t}\, \text{n}\text{e}\text{a}\text{r}\, \text{t}\text{h}\text{e}\, \text{n}\text{e}\text{w}\, \text{o}\text{b}\text{j}\text{e}\text{c}\text{t}-\text{o}\text{l}\text{d}\, \text{o}\text{b}\text{j}\text{e}\text{c}\text{t}}{\text{t}\text{i}\text{m}\text{e}\, \text{s}\text{p}\text{e}\text{n}\text{t}\, \text{n}\text{e}\text{a}\text{r}\, \text{t}\text{h}\text{e}\, \text{n}\text{e}\text{w}+\text{o}\text{l}\text{d}\, \text{o}\text{b}\text{j}\text{e}\text{c}\text{t}})$ which were analyzed using ethovision software. Rats with good cognitive function can remember both new and old objects and more interested for new object. Rats spend more time exploring familiar objects or there is no difference in exploration time, it can be interpreted as cognitive impairment in rats.

### Tissue preparation

On the 16th day post-injury, rats received intraperitoneal anesthesia with ketamine and xylazine, after which they were placed on a table with their heads securely held. Subsequently, a small incision was made in the skull, and the brain was carefully removed. Brain samples were placed in identical containers and fixed using 10% neutral buffered formalin. The containers were labeled with a combination of numbers and letters as a code. After 24 h, the brain tissue was sectioned coronally in the perilesional area to obtain cross-consecutive 5 μm tissue sections. The samples were dehydrated using a series of graded alcohol solutions and infiltrated with paraffin, then placed in paraffin molds and frozen. The paraffin blocks were then cut into thin sections, each mounted on a slide for hematoxylin-eosin staining and immunohistochemistry staining. The preparation of the slides was carried out by laboratory personnel specialized in anatomic pathology who were blinded to the experimental groups.

### GFAP immunohistochemistry

The mouse brain tissue, in the form of paraffin, was fixed in 10% buffered formalin for 24 h. Subsequently, deparaffinization and rehydration were performed by immersing the slides in xylene twice for 3 min each, followed by 100% ethanol for 3 min, and then washing in PBS for 5 min. The area around the slide sections was cleaned with a wipe, and the slides were immersed in a 3% hydrogen peroxide solution and absolute methanol for 15 min at room temperature. The slides were washed with PBS for 5 min, three times. The area around the slide sections was cleaned again with a wipe, then the primary antibody GFAP (2E1) Sc-33,673, Lot # 1022 Mouse monoclonal IgG2b (Santa Cruz Biotechnology) was added and incubated overnight at 4 °C. The slides were washed with PBS for 5 min, three times. Subsequently, the Avidin-Biotin Complex reagent Sc-516,216 (Santa Cruz Biotechnology) was added and incubated at room temperature for 30 min. The tissue sections were washed again with PBS for 5 min, three times. Next, 3,3’-diaminobenzidine chromogen (Nichirei Biosciences) was added and left at room temperature for 10 min. The slides were rinsed with distilled water for 5 min, three times. The slides were immersed in a counterstain solution and washed thoroughly with tap water. The final step involved mounting by placing the tissue sections on glass slides and covering them with permanent mounting media.

The formation of glial scars in this study was determined by (1) The expression of GFAP that is analogized with increased GFAP + cells; and (2) structural changes in astrocyte cells resulting in hypertrophy and proliferation, overlapping processes of astrocytes, loss of individual astrocyte domains, and the formation of a compact glial scar in the perilesional area through immunohistopathological examination by an expert in anatomic pathology. GFAP + cells was determined by counting the number of positive cells using a light binocular microscope with a magnification of 1000x in 20 fields of view in the perilesional area. Structural changes in astrocyte cells were observed by using a light binocular Olympus BX51 Microscope at a magnification of 1000x in the perilesional area. The observations were performed by an analyst who was blinded to the groups.

### Statistical analyses

The analysis of the weight drop model to elevate glial scar formation was conducted through Kruskal-Wallis and Mann-Whitney tests. To identify the weight drop model that leads to an increase in GFAP + cells and cognitive impairment, Shapiro-Wilk and Levene tests were performed, followed by one-way ANOVA and Bonferroni tests. Furthermore, the relationship between GFAP + cells and cognitive impairment was examined using the Pearson Correlation test.

## Result

This study observed the weight drop model and modifications in the height of the weight drop to investigate the formation of glial scars and cognitive impairments in each of the five rats in the treatment group. Throughout the experiment, no rats developed any illness or experienced mortality, ensuring that all rats remained included in the study without any dropouts.

### WD2 model elevates glial scar formation

The results of immunohistochemical staining were evaluated by experts blinded to the treatment group of the animals. The results were summarized into three groups based on the Sofroniew and Vinter theory, namely normal astrocytes, astrogliosis, and glial scar. In normal brain tissue, not all astrocytes expressed detectable levels of GFAP, had nonoverlapping domains, and showed little or no proliferation. In brain injuries with astrogliosis, most astrocytes are GFAP+, with disruptions of individual domains and proliferation. In brain injuries with the formation of glial scars, there were overlapping astrocytes, loss of individual domains, and evident dense, narrow, and clearly proliferating astrocytes in the perilesional area of the brain.

**Fig. 2:**
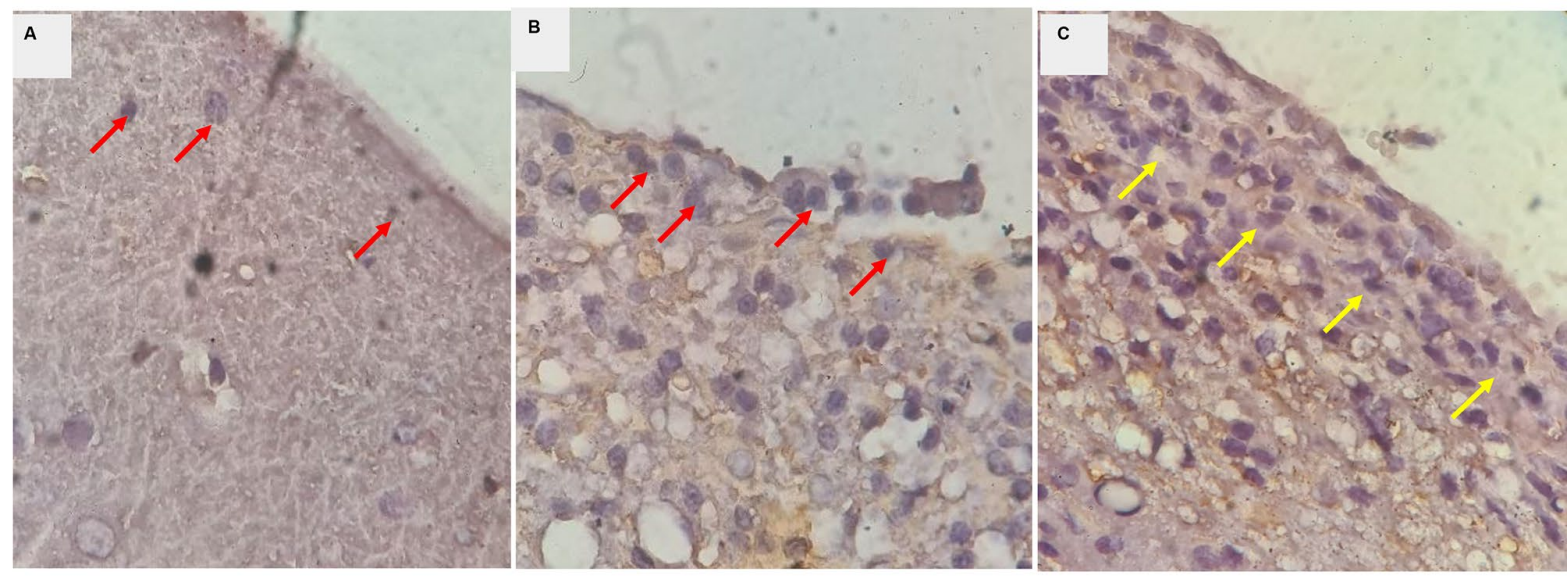
Representative histological changes through GFAP staining at 1000x magnification in perilesional area of the brain. Reactiedve astrocytes are indicated by brown uptake from GFAP and have blue-colored cell nuclei. (**A**) Normal astrocytes: Red arrows show that not all astrocytes express detectable levels of GFAP, astrocytes have non-overlapping domains, little or no proliferation; (**B**) Astrogliosis: Red arrows show features of astrocytes with increased GFAP + cells accompanied by hypertrophy, astrocyte proliferation, and loss of individual domains; and (**C**) Glial scar: Yellow arrows indicate the appearance of glial scar with more extensive upregulation of GFAP, dense, narrow, and well-defined hypertrophic astrocyte proliferation in the perilesional area

The staining results for GFAP in the healthy brain (sham group) depicted the presence of normal astrocytes (Fig. 2A). With mild trauma treatment, normal astrocytes transformed into active astrocytes or astrogliosis (Fig. 2B), which could resolve back into normal astrocytes. However, more severe trauma treatment induced the transformation of normal astrocytes into severe astrogliosis, persisting after two weeks in the perilesional area, indicating the formation of a glial scar (Fig. 2C) [11].

Based on the analysis of immunohistochemical staining results, there was no evidence of glial scar formation in the sham group. In the WD1 group, glial scars were found in 40.0% of the samples, while in the WD2 group, glial scars were found in 80.0% of the samples (Table 1). The Kruskal-Wallis test showed a significant difference between the sham, WD1, and WD2 groups, prompting further analysis using the Mann-Whitney test to determine specific comparisons between these groups.

**Table 1:** Results of Univariate Analysis of GFAP Immunohistochemistry Between Groups

Groups	Results			*p* value
	Normal Astrocytes	Astrogliosis	Glial scar	
Sham (a)	5 (100%)	0 (0%)	0 (0%)	a-b = 0.005*
WD 1 (b)	0 (0%)	3 (60.0%)	2 (40.0%)	b-c = 0.221
WD 2 (c)	0 (0%)	1 (20.0%)	4 (80.0%)	a-c = 0.004*

WD1, weight drop 1; WD2, weight drop 2; *shows a significant difference between groups (*p* < 0.05)

The WD1 and WD2 groups significantly induced the formation of glial scars compared to the sham group. This is supported by the significant differences observed between the sham and WD1 groups (*p* = 0.005) and between the sham and WD2 groups (*p* = 0.004), respectively (Table 1). Although there was no significant difference between the WD1 and WD2 groups, clinically, the WD2 group showed a higher prevalence of glial scar formation, as indicated in Table 1.

Trauma in WD1, induced by dropping a 10 g weight from a height of 5 cm, was found to cause normal astrocytes to become reactive. However, histopathologically, 60% formation of astrogliosis occurred, with only 40% progressing to form glial scars. On the other hand, in the WD2 group, trauma caused by dropping the same 10 g weight from a height of 10 cm led to more severe injuries, resulting in 80% glial scar formation and only 20% astrogliosis.

### WD2 model elevates GFAP + cells

The immunohistochemical results were observed by an analyst to quantify the GFAP + cells as a quantitative measure of glial scar formation. GFAP + cells was determined by counting the number of positive cells using a light binocular microscope with a magnification of 1000x in 20 fields of view in the perilesional area. One-way ANOVA test revealed a significant difference (*p* < 0.05) among the sham, WD1, and WD2 groups. Therefore, a post hoc Bonferroni’s test was conducted to determine which groups were significantly different from each other.

**Fig. 3:**
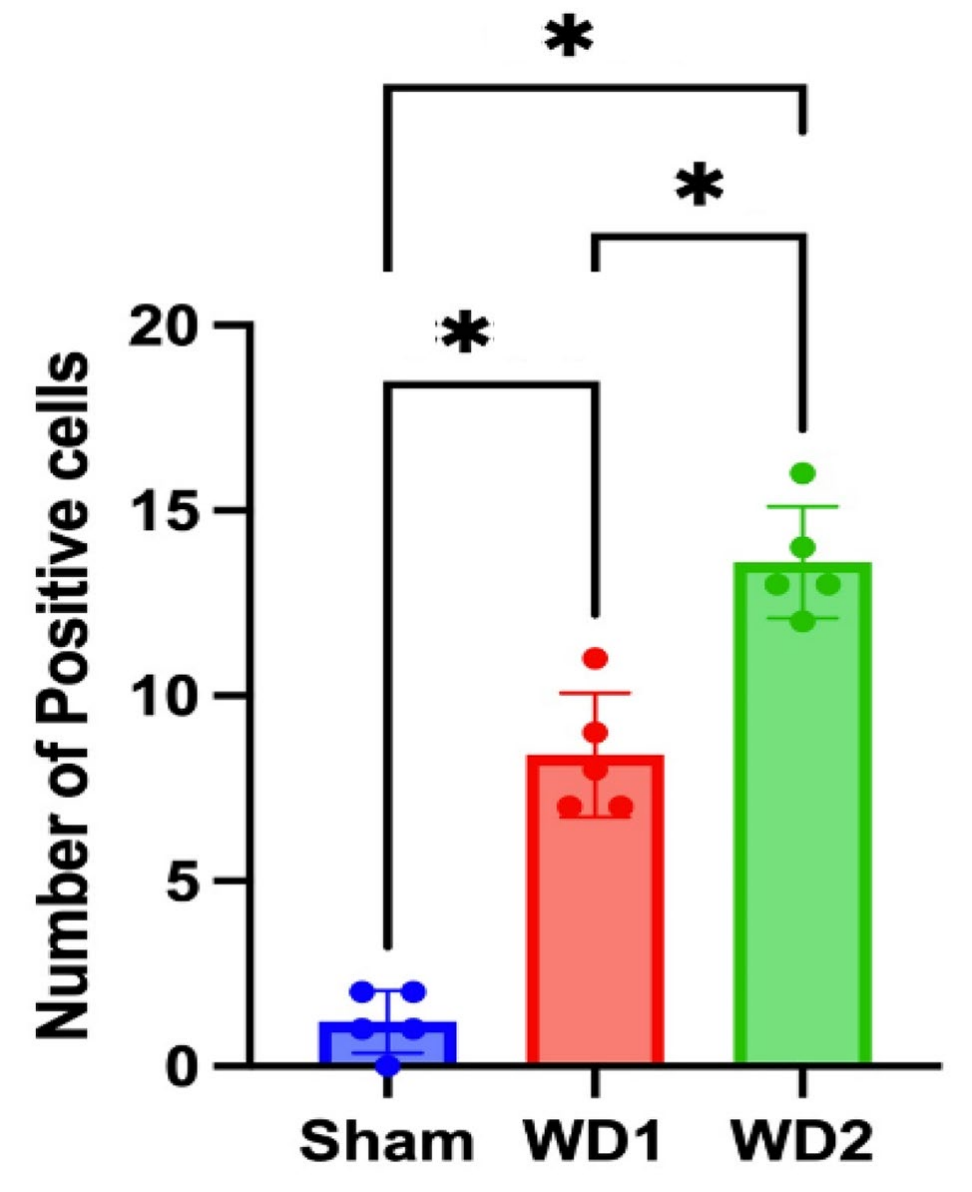
The weight drop model increased the GFAP + cells in the perilesional area of the brain. Data are presented as the mean ± standard deviation (n = 5 per group). *Indicates Bonferroni’s test results with *p* < 0.05

The post hoc Bonferroni’s test revealed significant differences (*P* = 0.0001) between the sham and WD1 groups, the sham and WD2 groups, and the WD1 and WD2 groups. Both the WD1 and WD2 groups exhibited the formation of glial scars; however, there was a statistically significant difference between the two groups. Clinically, WD2 showed worse results than WD1, as indicated by the mean values in Fig. 3.

### WD2 model decrease cognitive function

**Fig. 4:**
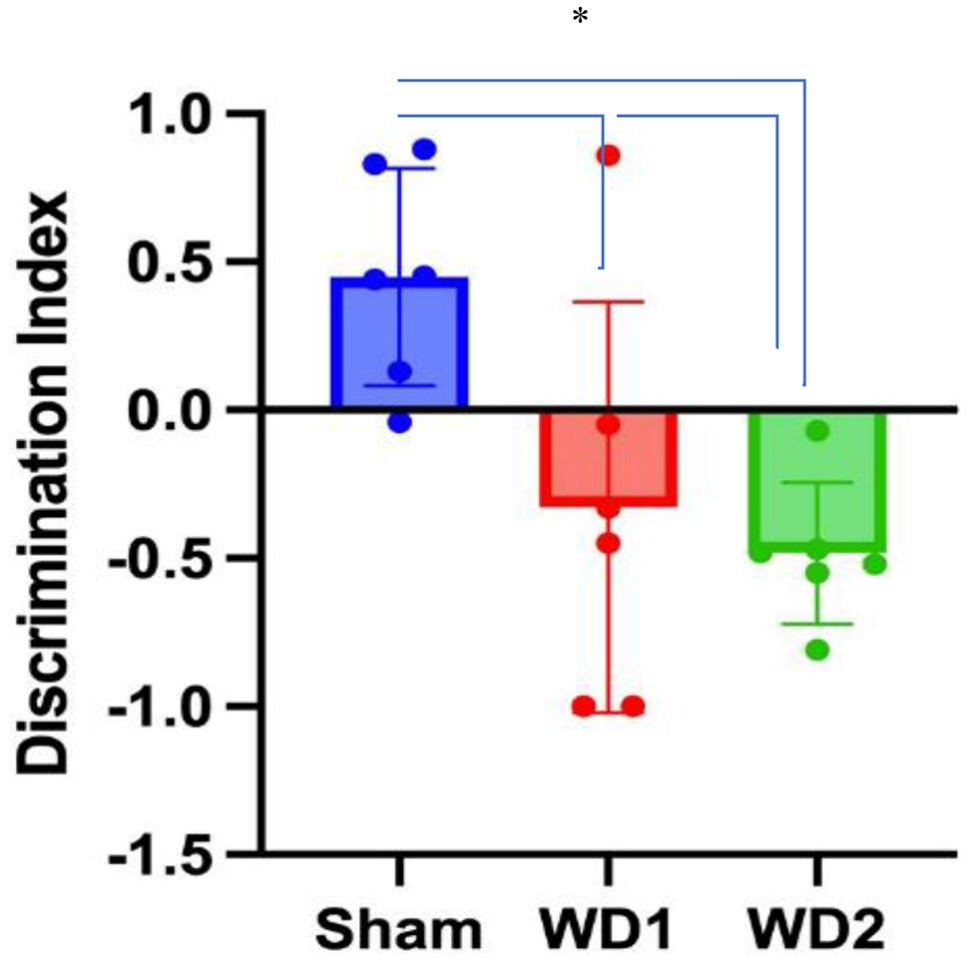
Modification of the height of WD model increased cognitive impairments assessed through the NOR test. Data are presented as the mean ± standard deviation (n = 5 per group). A low discrimination index indicates cognitive impairments

Cognitive assessment in rats using the NOR test can be observed using the discrimination index. Both WD1 and WD2 models resulted in rats exhibiting cognitive impairment, as observed from the mean values in Fig. 4. Meanwhile, statistically, the ANOVA test results showed a significant difference among the sham, WD1, and WD2 groups. Therefore, a post hoc Bonferroni’s test (homogeneous variance) was conducted to assess the group with the worst cognitive condition compared to the control group.

Based on the results of the post hoc Bonferroni’s test, only the WD2 group showed a significant difference compared to the sham group (*p* = 0.049), indicating that the WD2 group was the most effective in inducing cognitive impairment. Therefore, the WD2 model demonstrates a good discrimination index for distinguishing between healthy rats and rats with cognitive impairment.

### Increased GFAP + cells decreases cognitive function

The correlation analysis showed a significant relationship (*p* = 0,012) between GFAP + cells and the cognitive condition of rats. The negative correlation coefficient indicates that an increase in GFAP + cells will decrease the cognitive function of rats. The correlation coefficient value of 0.631 falls into the category of a moderate correlation between GFAP + cells and the cognitive condition of rats (Table 2). The more comprehensive statistical analysis results can be accessed in the supplementary file.

**Table 2:** The Correlations of GFAP + Cells with Cognitive Function

Results	Correlation coefficient		*P* Value
	GFAP + cells	Cognitive Function	
GFAP + cells	1	-0,631	0,012*
Cognitive Function	-0,631	1	

* shows a significant correlation (*p* < 0.05) between GFAP + cells and the cognitive function in all treatment groups (Sham, WD1, WD2)

## Discussion

The results of this study demonstrate that the weight drop model with the brain exposed facilitates researchers in confirming the occurrence of brain contusion, which is a prerequisite for the formation of glial scars. This can be observed through histopathological images with GFAP staining, indicating the presence of glial scars in the perilesional area of the brain. Although other studies have utilized the closed head injury method (with intact skin), the researchers did not use this method due to the higher risk of bias caused by variations in hair, skin thickness, and the rat skull, which could lead to concerns about significant variations in the formation of glial scars.

The formation of glial scars in this study was assessed through immunohistochemical examination, and the results revealed that both the WD1 and WD2 groups induced increased GFAP + cells compared to the sham group. Increasing GFAP + cells in the WD2 group showed worse results compared to the WD1 group. This finding is consistent with previous theories stating that the severity of trauma can influence the extent of astrogliosis in the process of glial scar formation. The increased GFAP + cells indicates the degree of severity of astrogliosis. Observation of the increased GFAP + cells for two weeks clearly indicated the formation of a glial scar [11].

In addition to the increased GFAP + cells, changes in the structure and morphology of astrocytes in the perilesional area can support the definition of glial scar formation according to Sofroniew and Vinters. Histopathological observations of glial scars reveal hypertrophy and proliferation of astrocytes, overlapping processes of astrocytes, loss of individual astrocytic domains, and the formation of compact glial scars in the perilesional area of the brain. In this study, it was found that both the WD1 and WD2 groups induced significant formation of glial scars compared to the sham group. However, clinically, the WD2 group showed the highest extent of glial scar formation, supporting the previous theory that the severity of injury correlates with the histopathological manifestation of glial scar formation.

This study on TBI models utilized the weight drop method on the exposed right frontal brain, which has been proven to induce cognitive impairments assessed through the NOR test. Through the calculation of the discrimination index, both WD1 and WD2 models equally induced cognitive impairments in rats, as indicated by their preference for exploring familiar objects over novel ones. However, statistical analysis revealed that only the WD2 model resulted in the most severe cognitive impairment. This suggests that the method employed in the WD2 model is the most effective in inducing cognitive impairments in rats with TBI. Cognitive impairments such as memory deficits, attention problems, poor reasoning, and dysprosodies can occur due to brain damage in the right frontal lobe, as observed in this study [14]. The NOR test is widely used as a tool for assessing visual memory in rodents due to its convenient administration and short testing time [7]. The ability of the NOR test to assess cognitive impairment in rats subjected to TBI has been demonstrated in various previous studies, including Qubty et al. [15] Stetter et al., [16] Sekar et al., [17] Kempuraj et al., [18] and Chen et al. [13]

In this study, cognitive function was observed up to day 14 after injury, and persistent cognitive impairments were observed. Persistent cognitive impairments are theoretically caused by the formation of glial scars, which obstruct axonal repair and neuronal function recovery in the CNS and induce the release of abnormal molecular mechanisms in the late stage of TBI [10]. The WD2 group is the most effective model in inducing the formation of glial scars and cognitive impairments. This weight drop model utilizes a 10 g weight dropped from a height of 10 cm onto the right exposed brain of Sprague Dawley rats. Glial scar formation was observed two weeks after injury. The diagnosis of glial scar was established using criteria by Sofroniew and Vinters, through immunohistopathological observation by an expert anatomical pathologist and by counting the expression of astrocytes through GFAP staining. This TBI model in the study also demonstrated persistent cognitive impairments two weeks after injury, which were assessed through the NOR test. From this study, it can be concluded that an increase in GFAP + cells, as one of the indicators of glial scar formation, can impair cognitive function in experimental animals. Therefore, inhibiting glial scar formation could be a potential strategy to enhance cognitive function in experimental animals following TBI.

Previously, there have been no specific studies on a weight drop model that specifically addresses the induction of glial scar formation and cognitive impairments. However, there are several studies, such as Chen and Luo, that discuss the relationship between TBI and behavioral deficits while indirectly observing astrocyte activity. In the study by Chen et al., [13] which focuses on the relationship between TBI, acute behavioral deficits, and mitochondrial changes, the weight drop model was performed by dropping a 10 g weight from a height of 5 cm onto the exposed right brain of Sprague Dawley rats. This model demonstrated cognitive impairments through NOR examination at 1 day and 1 week after injury and confirmed an increase in astrogliosis in the cortex, hippocampus, and thalamus at 1 day and 6 weeks after injury. In the study by Luo et al. [19] on neural stem cells (NSC) transplantation to improve cognitive function recovery in rats with TBI, the weight drop model was performed by dropping a 10 g weight at a speed of 4 m/s onto the exposed right brain of Sprague Dawley rats. This study revealed cognitive impairments on the fifth day after injury, as assessed through the MWM test. On the 14th day after injury, glial scar formation was observed in the group that received the weight drop model. Although the two previous studies did not directly discuss glial scar formation, they served as references for researchers to modify the weight drop model according to the objectives of this study.

By adjusting the height of the weight drop model to 10 cm using the same method, the study might clarify previous findings by providing results of glial scar formation in rats with traumatic brain injury. It is hoped that this modification of the weight drop model can serve as a reference for further research on drugs or other substances that can inhibit glial scar formation and improve cognitive impairments. Nonetheless, it is important to acknowledge several limitations within this study. Firstly, variations of the weight drop model was exclusively executed with heights of 5 and 10 cm. Further research is needed to explore other variations in weight and drop height. Additionally, the control group in this study did not undergo surgery and brain exposure because this method is very difficult to perform. The risk of unintended injury is a concern that could lead to a biased representation of glial scar on the surface of the brain in the control group. In the weight drop (WD) group, the brain exposure method was performed by opening the skull while preserving the brain’s protective layer (dura mater) to prevent brain damage and ensure that brain function is not disrupted. Furthermore, cognitive function in rats was evaluated through the NOR test, so the evaluation of other neurobehavioral impairments using various tools such as Morris Water Maze and Y Maze is needed. To comprehensively analyze the glial scar, it is imperative to conduct the quantification of reactive astrocyte cell counts within the scar through the assessment of GFAP intensity using fluorescence. Apart from observing the physical formation of the glial scar, it is also important to examine the production of proteins, including CSPG which molecularly inhibit axonal regeneration.

## Conclusion

In summary, the modification of the height of the weight drop model by dropping a weight of 10 g from a height of 10 cm onto the right brain exposed of the rat has been proven to induce the formation of a glial scar and leads to cognitive impairments.

### Electronic supplementary material

Below is the link to the electronic supplementary material.

Supplementary Material 1

## Data Availability

The data used in the article is presented in the article/supplementary material. Please contact the corresponding author for further information.
