# Supplementary material for: Modification of the height of a weight drop traumatic brain injury model that causes the formation of glial scar and cognitive impairment in rats
Source: BMC Neurol. 2023 Dec 15;23:439. doi: 10.1186/s12883-023-03494-y (PMC10722700; doi:10.1186/s12883-023-03494-y)
Supplement: Supplementary file 1 — Supplementary Material 1 [file 12883_2023_3494_MOESM1_ESM.docx]

**WD2 Models Elevates Glial Scar Formation**

Table S1. Kruskal Wallis Test Result

| Groups | Results | | | Total | P value |
| --- | --- | --- | --- | --- | --- |
|  | Normal Astrocytes | Astrogliosis | Glial scar |  |  |
| Sham | 5 (100%) | 0 (0%) | 0 (0%) | 5 (100%) | 0.002* |
| WD 1 | 0 (0%) | 3 (60.0%) | 2 (40.0%) | 5 (100%) |  |
| WD 2 | 0 (0%) | 1 (20.0%) | 4 (80.0%) | 5 (100%) |  |

Table S2. Mann Whitney Test Result

| Sham | P value |
| --- | --- |
| Sham – WD 1 | 0,005* |
| Sham – WD 2 | 0,004* |
| WD 1 – WD 2 | 0,221 |

**WD2 Model Elevates GFAP+ Cells**

Table S3. The number of astrocyte among groups

| Rats Sequence | Groups | | |
| --- | --- | --- | --- |
|  | Sham | WD1 | WD2 |
| 1 | 2 | 8 | 13 |
| 2 | 1 | 7 | 12 |
| 3 | 0 | 9 | 14 |
| 4 | 1 | 7 | 13 |
| 5 | 2 | 11 | 16 |
| Mean | 1.2 | 8.4 | 13.6 |

Table S4. Normality testing using the Shapiro Wilk test (n<50)

| Groups | n | Shapiro Wilk | Results |
| --- | --- | --- | --- |
| Sham | 5 | 0.314 | Normal |
| WD 1 | 5 | 0.314 | Normal |
| WD 2 | 5 | 0.492 | Normal |

Table S5. Homogeneity testing using the Levene test

| Groups | Levene | Result |
| --- | --- | --- |
| Sham | 0.225 | Homogeneous |
| WD 1 |  |  |
| WD 2 |  |  |

Table S6. One Way Anova Test Results

| Groups | n | Mean | SD | P value |
| --- | --- | --- | --- | --- |
| Sham | 5 | 1.20 | 0.837 | 0.0001* |
| WD 1 | 5 | 8.40 | 1.673 |  |
| WD 2 | 5 | 13.60 | 5.418 |  |

Table S7. Bonferrroni Test Results

| Groups | P value |
| --- | --- |
| Sham – WD 1 | 0.0001* |
| Sham – WD 2 | 0.0001* |
| WD 1 – WD 2 | 0.0001* |

**WD2 Model Decreases Cognitive Function**

Table S8. Discrimination index data

| Rats Sequence | Groups | | |
| --- | --- | --- | --- |
|  | Sham | WD1 | WD2 |
| 1 | 0.13 | 0.86 | -0.81 |
| 2 | -0.04 | -1 | -0.07 |
| 3 | 0.44 | -1 | -0.47 |
| 4 | 0.83 | -0.05 | -0.55 |
| 5 | 0.88 | -0.45 | -0.52 |
| Mean | 0.45 | -0.33 | -0.48 |

Table S9. Normality testing using the Shapiro Wilk test (n<50)

| Groups | n | Shapiro Wilk | Results |
| --- | --- | --- | --- |
| Sham | 5 | 0.439 | Normal |
| WD 1 | 5 | 0.364 | Normal |
| WD 2 | 5 | 0.502 | Normal |

Table S10. Anova Test Results

| Groups | n | Mean | SD | P value |
| --- | --- | --- | --- | --- |
| Sham | 5 | 0.45 | 0.41 | 0.036* |
| WD 1 | 5 | -0.33 | 0.78 |  |
| WD 2 | 5 | -0.48 | 0.27 |  |

Table S11. Bonferroni Post Hoc Testing Results (homogeneous variance)

| Groups | P Value |
| --- | --- |
| Sham – WD 1 | 0.116 |
| Sham – WD 2 | 0.049* |
| WD 1 – WD 2 | 1.000 |

**Increased GFAP+ Cells Decreases Cognitive Function**

Table S12. Raw data on the correlation of GFAP expression with cognitive impairment

| Rats Sequence | Discrimination Index | GFAP Expression |
| --- | --- | --- |
|  | Sham | |
| 1 | 0.127137499 | 2 |
| 2 | -0.036772255 | 1 |
| 3 | 0.443200546 | 0 |
| 4 | 0.832765525 | 1 |
| 5 | 0.883810188 | 2 |
| Rats Sequence | WD 1 | |
| 1 | 0.863563168 | 8 |
| 2 | -1 | 7 |
| 3 | -1 | 9 |
| 4 | -0.055304636 | 7 |
| 5 | -0.454049136 | 11 |
| Rats Sequence | WD2 | |
| 1 | -0.814747997 | 13 |
| 2 | -0.066003249 | 12 |
| 3 | -0.475304738 | 14 |
| 4 | -0.546251376 | 13 |
| 5 | -0.520763902 | 16 |

Table S13. Normality testing using the Shapiro Wilk test (n<50)

| Variable | Groups | n | Saphiro Wilk | Results |
| --- | --- | --- | --- | --- |
| Cognitive Impairment | Sham | 5 | 0,438 | Normal |
|  | WD 1 | 5 | 0,364 | Normal |
|  | WD 2 | 5 | 0,503 | Normal |
| GFAP Expression | Sham | 5 | 0,314 | Normal |
|  | WD 1 | 5 | 0,314 | Normal |
|  | WD 2 | 5 | 0,492 | Normal |
